# Screen-detected breast cancers have a lower mitotic activity index

**DOI:** 10.1054/bjoc.1999.0930

**Published:** 2000-01-18

**Authors:** R P R Groenendijk, P Bult, L Tewarie, P G M Peer, R F van der Sluis, T J M Ruers, T Wobbes

**Affiliations:** Departments of Surgery, Pathology, Medical Statistics, University Hospital Nijmegen Sint Radboud, PO Box 9101, Nijmegen, 6500, The Netherlands

**Keywords:** breast, cancer, screening mitotic, activity, index

## Abstract

We know that screening for breast cancer leads to detection of smaller tumours with less lymph node metastases. Could it be possible that the decrease in mortality after screening is not only caused by this earlier stage, but also by a different mitotic activity index (MAI) of the tumours that are detected by screening? Is MAI a prognostic factor for recurrence-free survival? A retrospective study was carried out of 387 patients with breast cancer, treated at the University Hospital Nijmegen between January 1992 and September 1997. Ninety patients had screen-detected breast cancer, 297 patients had breast cancers detected outside the screening programme. The MAI, other prognostic factors and recurrence-free survival were determined. In non-screen-detected tumours the MAI is twice as high as in screen-detected tumours, even after correction for age took place. The MAI correlated well with other tumour characteristics. The MAI in itself is a prognostic factor for recurrence-free survival. Favourable outcome in screen detected breast cancer is not entirely caused by detecting cancer in early stages: quantitative features such as the MAI indicate a less malignant character of screen detected breast cancer. The MAI is an independent prognostic factor for recurrence-free survival. © 2000 Cancer Research Campaign


*British Journal of Cancer* (2000) **82**(2), 381–384
© 2000 Cancer Research Campaign
Article no. bjoc.1999.0930

### Screen-detected breast cancers have a lower mitotic activity index

**RPR Groenendijk[1], P Bult[2], L Tewarie[1], PGM Peer[3], RF van der Sluis[1], TJM Ruers[1] and T Wobbes[1]**

Departments of [1]Surgery, [2]Pathology and [3]Medical Statistics, University Hospital Nijmegen Sint Radboud, PO Box 9101, 6500 HB Nijmegen The Netherlands

**Summary** We know that screening for breast cancer leads to detection of smaller tumours with less lymph node metastases. Could it be possible that the decrease in mortality after screening is not only caused by this earlier stage, but also by a different mitotic activity index (MAI) of the tumours that are detected by screening? Is MAI a prognostic factor for recurrence-free survival? A retrospective study was carried out of 387 patients with breast cancer, treated at the University Hospital Nijmegen between January 1992 and September 1997. Ninety patients had screen-detected breast cancer, 297 patients had breast cancers detected outside the screening programme. The MAI, other prognostic factors and recurrence-free survival were determined. In non-screen-detected tumours the MAI is twice as high as in screen-detected tumours, even after correction for age took place. The MAI correlated well with other tumour characteristics. The MAI in itself is a prognostic factor for recurrence-free survival. Favourable outcome in screen detected breast cancer is not entirely caused by detecting cancer in early stages: quantitative features such as the MAI indicate a less malignant character of screen detected breast cancer. The MAI is an independent prognostic factor for recurrence-free survival. © 2000 Cancer Research Campaign

**Keywords:** breast; cancer; screening mitotic; activity; index

Screen-detected breast cancer patients show a better survival than breast cancer patients who are detected outside a screening programme (Wolfe, 1991; Harris and Vogel, 1997; Schroën et al, 1998). According to the early results of the Dutch trial in Nijmegen the breast cancer mortality rate in women of 35 and over can be reduced by roughly 50% by regular mammographic screening of all eligible women (Verbeek et al, 1984). Other studies have shown similar results (Tabàr, 1985; Peeters et al, 1998). This is mainly explained by early detection of screen-detected tumours. It may, however, be possible that factors other than tumour size are responsible for better survival figures. Screen-detected cancers may be less aggressive than non-screen-detected cancers.

One way to determine tumour aggressiveness is to measure the mitotic activity index (MAI). The MAI is an index made out of the mitotic frequency in the most active part of the tumour, and therefore is a quantitative feature of the tumour. In recent literature quantitative factors, such as the MAI, are good prognostic factors for patient survival (Baak, 1985; Baak et al, 1985; van der Linden et al, 1986, 1987, 1989; Theissig et al, 1996; Uyterlinde et al, 1987, 1990; Aaltomaa et al, 1991; van Diest et al, 1992). They discriminate very well less aggressive tumours from more aggressive ones. Measuring the MAI is easily learned and can be performed in a highly reproducible way if a strict protocol is carefully followed (van Diest et al, 1992; Jannink et al, 1995; Collan et al, 1996).

The aim of this study was to investigate whether there is a difference in the MAI between screen and non-screen detected breast cancers, and to evaluate the prognostic value of the MAI for recurrence free survival, adjusting for other prognostic factors.

*Received 8 March 1999*
*Revised 28 July 1999*
*Accepted 3 August 1999*

*Correspondence to:* RPR Groenendijk

### MATERIALS AND METHODS

A retrospective evaluation was performed of 387 evaluable patients with breast cancer who were treated between January 1992 and September 1997. The median period of follow up was 2.6 years (range 0.2–6.2). Ninety patients with a mean age of 58 years (range 48–72 years), had screen-detected breast cancer, while 297 patients with a mean age of 54 years (range 28–93 years), had non-screen-detected breast cancer. Breast cancer screening was usually performed in the age group 50–70 years. Patients were surgically treated with a modified radical mastectomy ($n$ = 305, 79%) or a breast saving procedure ($n$ = 82, 21%). Radiotherapy was used in breast saving procedures (21%) and in case of T3/T4 tumours or chest-wall contamination (11%). Adjuvant irradiation of the axilla was used when there was extranodal involvement (15%). Radiotherapy was used in screen- and non-screen-detected patients in the same frequency. Use of adjuvant chemotherapy was depending on lymph node involvement and menopausal status. Chemotherapy was given as CMF (cyclophosphamide, 100 mg $m^{-2}$ orally on days 1–14; methotrexate, 40 mg $m^{-2}$ intravenously on days 1 and 8; and fluorouracil, 600 mg $m^{-2}$ intravenously on days 1 and 8) in 105 patients (27%). Use of adjuvant chemotherapy was not different for screen- or non-screen-detected patients. Hormone treatment in the form of adjuvant tamoxifen 20 mg $day^{-1}$ for a period of 5 years was given to 79 patients (20%), according to receptor status, menopausal status and lymph node status. Use of hormone treatment was equal over groups of screen- and non-screen-detected patients. Tumour size, histological type, differentiation grade, hormone receptor status and lymph node involvement were determined by the pathologist. Grading of the invasive carcinoma was carried out according to the Elston method described in Diagnostic Histopathology of the Breast by Page and Anderson (Elston, 1987). The method involves the assessment of three components of tumour morphology: tubule formation, nuclear pleomorphism

**Table 1** Patient characteristics of 176 patients between 50 and 70 years

| | Screen-detected | Non-screen-detected | |
|---|---|---|---|
| Number | 82 | 94 | |
| Mean age | 58 (50–70) | 58 (50–70) | NS |
| Follow-up (years) | 2.7 (0.3–5.9) | 2.7 (0.3–5.9) | NS |
| MAI (median) | 6 | 12 | $P < 0.001$ |
| Tumour status | | | |
| T1 | 51/82 | 35/94 | |
| T2 | 27/82 | 38/94 | |
| T3 | 1/82 | 9/94 | |
| T4 | 3/82 | 12/94 | |
| N0 | 56/82 | 38/94 | |
| N1 | 24/82 | 48/94 | |
| N2 | 2/82 | 3/94 | |
| Unknown | 0/82 | 5/94 | |
| Mastectomy | 55/82 | 77/94 | NS |
| Adjuvant treatment | | | |
| Chemotherapy | 11/82 | 18/94 | NS |
| Hormone therapy | 18/82 | 25/94 | NS |
| Radiotherapy | 32/82 | 50/94 | NS |

NS, not significant.

**Table 2** MAI for screen- and non-screen-detected cancers, stratified for T and N stages, all patients between 50 and 70 years

| | MAI geometric mean ($P_{25}$-$P_{75}$) | | *P*-value |
|---|---|---|---|
| | Screen-detected | Non-screen-detected | |
| Overall (all ages) | 6 (3–16) *n* = 90 | 12 (6–29) *n* = 297 | < 0.0001 |
| Tumour size | | | |
| T1 | 6(3–15) *n* = 51 | 12(6–36) *n* = 35 | = 0.004 |
| T2 | 6(2–19) *n* = 27 | 14(6–27) *n* = 38 | = 0.03 |
| Nodal status | | | |
| N0 | 5(2–13) *n* = 56 | 11(5–23) *n* = 38 | = 0.0009 |
| N1 | 9(5–20) *n* = 24 | 12(6–29) *n* = 48 | = 0.28 (NS) |

NS, not significant; *n*, number of tumours.

and frequency of mitoses. Tubule formation was scored as 1 if the great majority of the tumour was composed of formed tubules. When tubule formation was seen in moderate amounts, a score of 2 points was made. Where little or no tubule formation was seen, the score was given as 3 points. For nuclear pleomorphism a score of 1, 2, or 3 was given if the tumour cells showed little, moderate or strong variation in shape and size of the nuclei respectively. The mitotic rate was assessed by counting the number of mitoses in ten high-power fields. Fewer than ten mitoses were given 1 point, 10–19 mitoses were given 2 points, and 20 mitoses or more were given 3 points. Adding the three scores together gave the histological grade of differentiation. Tumours were considered well differentiated (grade I) if the total score was 3–5 points ($n$ = 76, 19%), moderately differentiated (grade II) with a score of 6–7 points ($n$ = 157, 40%), and poorly differentiated (grade III) if the score was 8–9 points ($n$ = 161, 41%). The MAI was assessed in the subjectively most cellular and proliferative area of the tumour, usually at the periphery of the tumour, avoiding regions of necrosis and inflammation. The mitosis counting was performed using a Leitz microscope at ×400 magnification (×10 ocular and ×40 objective with a numerical aperture of 0.70 and a field diameter of 525 μm). The MAI was determined by counting the mitoses in ten consecutive high-power fields (Jannink et al, 1995).

### Statistical methods

Differences in geometric means of the MAI between groups were tested for significance after logarithmic transformation of the MAI. This was performed with a one-way analysis or a *t*-test depending on the number of groups. The difference in MAI between screen-detected and non-screen-detected cancers was adjusted for other tumour characteristics, i.e. tumour size, lymph node involvement, lymph vessel invasion, blood vessel invasion and hormone receptor status by multiple regression analysis.

Recurrence-free actuarial survival curves were computed for tumours with MAI from 1 to 9, 10–24 and above 24. The relation between the MAI and recurrence free survival was adjusted for other tumour characteristics and detection mode by a proportional hazards model. A significance level of 0.05 was used.

### RESULTS

In patients who were not screen-detected ($n$ = 297), there was no difference in the MAI between 148 patients younger than 50 years and 149 patients of 50 years or older.

Because the patients in the non-screen-detected group were younger, the non-screen-detected group was matched for age with the screen-detected group by selecting all patients from 50 to 70 years old. We chose this group because in the screening group the majority of patients were between 50 and 70 years (82/90). After adjusting for age 176 of 387 screen- and non-screen-detected patients remained between 50 and 70 years old. The patient characteristics of these groups are given in Table 1. In this group the ratio of the geometric means of the MAI in the non-screen-detected versus screen-detected remained 2.0 (confidence interval (CI) 1.4–2.8, $P < 0.0001$). The geometric mean of the MAI in the screen-detected group was half the geometric mean of the non-screen-detected group (6 vs 12, $P < 0.0001$), even after stratification for T and N stages. Only in N1 patients from 50 to 70 years was the MAI not significantly different (Table 2).

The MAI correlated well with other important tumour factors, such as histological type, lymph vessel invasion, blood vessel invasion, tumour size and lymph node involvement, and receptor status (Table 3).

After adjustment for mode of detection, tumour stage (T), lymph node involvement (N), lymph vessel invasion, blood vessel invasion and hormone recptor status in a multivariate analysis, the MAI remained a strong independent prognostic factor for recurrence of disease. The risk ratio for recurrence of disease after adjustment for all variables in a multivariate analysis was 1.01 ($P < 0.04$).

After adjustment for T, N, lymph vessel invasion, and blood vessel invasion in a multivariate analysis of all patients between 50 and 70 years, the ratio of the geometric means of the MAI was 1.7 (CI 1.2–2.4, $P < 0.0004$).

After adjustment for hormone receptor status, the ratio was 1.4 (CI 0.95–1.9, $P < 0.09$), indicating that even after correction for these prognostic factors, the MAI is higher in the non-screen detected than in the screen detected patients with breast cancer.

© 2000 Cancer Research Campaign

**Table 3** Relation of the MAI with other prognostic factors

| Factors | *n* | MAI geometric mean | *P*-value |
|---|---|---|---|
| Histological type | | | |
| Ductal | 272 | 13 | |
| Lobular | 53 | 4 | |
| Tubular | 12 | 1 | |
| Medullar | 6 | 45 | |
| Mixed type | 36 | 8 | 0.001 |
| Rest | 8 | | |
| Lymph vessel invasion | | | |
| Positive | 195 | 7 | |
| Negative | 190 | 16 | <0.001 |
| Blood vessel-invasion | | | |
| Positive | 24 | 20 | |
| Negative | 361 | 10 | 0.007 |
| T-stage | | | |
| T1 | 153 | 8 | |
| T2 | 145 | 12 | |
| T3 | 33 | 15 | |
| T4 | 51 | 13 | 0.005 |
| N-stage (nodal status) | | | |
| N0 | 180 | 8 | |
| N1 | 187 | 13 | |
| N2 | 7 | 20 | <0.001 |
| Oestrogen receptor status | | | |
| Positive | 25 | 9 | |
| Negative | 82 | 18 | <0.001 |
| Progesterone receptor status | | | |
| Positive | 26 | 9 | |
| Negative | 100 | 20 | 0.002 |

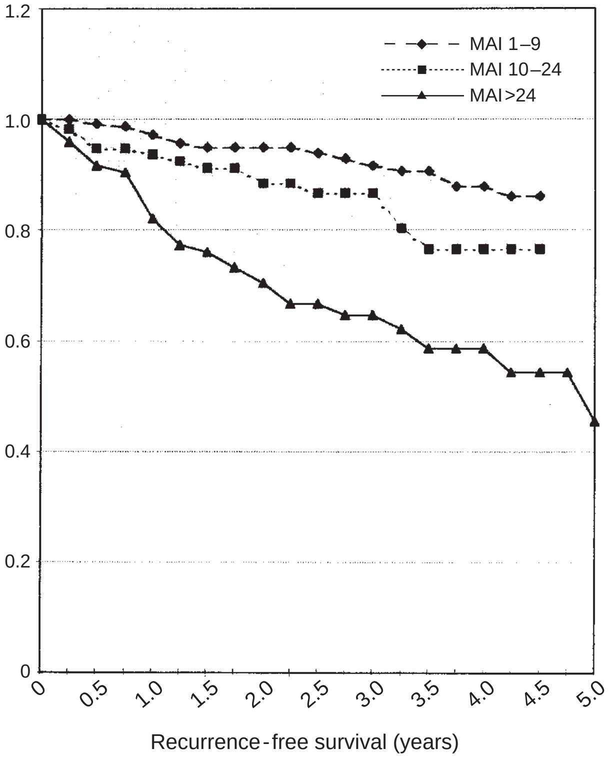

**Figure 1** Recurrence-free survival for tumours with a MAI from 1–9, 10–24, and above 24. Log rank test: *P*<0.0001.

The 5-year recurrence-free survival was 90% in the screen-detected group, versus 67% in the other group. Also the MAI was a significant univariate prognostic factor for recurrence-free survival (Figure 1).

### DISCUSSION

Screening for breast cancer is an effective method to detect breast cancer in an early stage of disease. Clinical breast examination and mammography are recommended as combined modalities for breast cancer screening (Freund et al, 1998). The use of supplemental ultrasound has been advocated in patients with radiographically dense breasts, but there appears to be no significant contribution to the accuracy of the work-up (Maestro et al, 1998). In The Netherlands the screening programme has been introduced in the whole country for women between 50 and 70 years old (Fracheboud et al, 1998). Population survey in younger women seems to be less beneficial (Smart, 1992; Elwood et al, 1993; Peer et al, 1996; Harris and Vogel, 1997). Recently the age for screening in The Netherlands has been increased up to 75 years, because a reduction in breast cancer mortality due to mammographic screening has been shown for women aged up to 75 years (van Dijck et al, 1997*a*, 1997*b*). A breast cancer screening programme leads to an increased use of breast conserving therapy and an increased need for post-operative radiotherapy; there will also be a higher number of women diagnosed with non-invasive breast cancer (Borras et al, 1998). As we have shown both in this study and before (Schroën et al, 1996), the screen-detected tumours were more often in an earlier stage than the tumours detected outside the screening programme. The recurrence-free survival in patients with tumours detected in the screening programme, was significantly better compared to patients with tumours detected outside the screening programme. The finding that the MAI was low in the screen-detected group supports the idea that screen-detected tumours are generally more favourable than others, because they are growing more slowly and are detected with mammography earlier for that reason. We considered only the MAI and not other factors that contribute to differentiation grade of the tumour. The length time bias caused by this phenomenon has always been a point of controversy in uncontrolled breast screening projects. Our study makes clear that this bias is certainly not only of theoretical importance. That the low MAI is found not only in T1 tumours, but also in T2 indicates that T2 tumours detected outside the screening programmes are of a different biological behaviour. The large size of the tumour is per se not a prognostic factor but the MAI of it is.

The MAI itself is an important prognostic factor for recurrence free survival. We were able to corroborate other studies that the MAI correlates well to other prognostic factors. The prognostic importance is even better when the MAI is used in other scoring systems, such as the multivariate prognostic index (MPI), which combines the MAI, lymph node status and tumour size (van der Linden et al, 1987). We found that the MAI alone is an important predictor for disease-free survival.

The MAI is a factor that can attribute to predicting poor responders to chemotherapy (van Diest et al, 1992). Others suggested that the decisions on adjuvant therapy in breast cancer can be based on the MAI and the MPI, particularly in node-negative patients (Baak et al, 1989, 1993; Aaltomaa et al, 1993). Assessing morphometric features of a resected specimen of breast cancer should be incorporated in routine pathology reports (van der Linden et al, 1987; Baak et al, 1992). Regional differences in prognosis of breast cancer are correlated with the MAI and other microscopic features (Baak et al, 1992). According to Jannink et al (1995) counting the MAI as we described before gives the strongest prognostic value. Collan et al give an efficient description of the method and its reliability (Collan et al, 1996). It takes about 10–15 min extra time to calculate the MAI in a tumour. It can be learned within a reasonable time to perform mitosis counting in a highly reproducible manner in a routine setting. However, motivation and on-going quality control are essential to guarantee the reproducibility of the assessments (van Diest et al, 1992; Uyterlinde et al, 1995).

As far as we know there is no previous study investigating the MAI in relation to the mode of detection of breast cancer. Tumours detected by screening have a significant lower MAI than tumours detected outside the screening programme. This implies that these tumours have a different profile, even when they have the same stage of disease. Besides the fact that screen detected tumours are detected earlier, they are also equipped with a favourable MAI, thus leading to a better prognosis.

### REFERENCES

Aaltomaa S, Lipponen P, Eskelinen M, Alhave E and Syrjänen K (1991) Nuclear morphometry and mitotic indexes as prognostic factors in breast cancer. *Eur J Surg* **157:** 319–324

Aaltomaa S, Lipponen P, Eskelinen M, Kosma VM, Marin S, Alhava E and Syrjänen K (1993) Predictive value of a morphometric prognostic index in female breast cancer. *Oncology* **50:** 57–62

Baak JPA (1985) The relative prognostic significance of nucleolar morphometry in invasive ductal breast cancer. *Histopathology* **9**: 437–444

Baak JPA, Dop van H, Kurver PHJ and Hermans J (1985) The value of morphometry to classic prognosticators in breast cancer. *Cancer* **56**: 374–382

Baak JPA et al (1989) The multicenter morphometric mammary carcinoma project (MMMCP): a nation wide prospective study on reproducibility and prognostic power of routine quantitative assessments in the Netherlands. *Pathol Res Pract* **185**: 664–670

Baak JPA, Wisse-Brekelmans ECM, Kurver PHJ, Gorp van LHM, Voorhorst FJ and Miettinen BS (1992) Regional differences in breast cancer survival are correlated with differences in differentiation and rate of proliferation. *Hum Pathol* **23**: 989–992

Baak JPA, Diest van PJ, Benraadt T, Matze-Cok E, Brugghe J, Schuurmans LT, Littooy JJ and other MMMCP collaborators (1993) The multi-center morphometric mammary carcinoma project (MMMCP) in the Netherlands: value of morphometrically assessed proliferation and differentiation. *J Cell Biochem Suppl* **17G**: 220–225

Borras JM, Espinas JA, Beemsterboer PM, Granados A and Koning de HJ (1998) Anticipating the consequences for the primary therapy of breast cancer after introducing screening. A more global picture for health care policy making. *Int J Technol Assess Health Care* **14**: 268–276

Collan YUI et al (1996) Standardized mitotic counts in breast cancer. Evaluation of the method. *Pathol Res Pract* **192**: 931–941

Diest van PJ et al (1992*a*) Reproducibility of mitosis counting in 2,469 breast cancer specimens: results from the multicenter morphometric mammary carcinoma project. *Hum Pathol* **6**: 603–607

Diest van PJ, Fleege JC and Baak JPA (1992*b*) Syntactic structure analysis in invasive breast cancer: analysis of reproducibility, biologic background and prognostic value. *Hum Pathol* **23**: 876–883

Diest van PJ, Baak JPA, Matze-Cok P and Bacus SS (1992*c*) Prediction of response to adjuvant chemotherapy in premenopausal lymph node positive breast cancer patients with morphometry, DNA flow cytometry and Her-2/Neu oncoprotein expression. *Pathol Res Pract* **188**: 344–349

Dijck van JA, Broeders MJ and Verbeek AL (1997*a*) Mammographic screening in older women. Is it worthwhile? *Drugs Aging* **10**: 69–79

Dijck van JA, Verbeek AL, Beex LV, Hendriks JH, Holland R, Mravunac M, Straatman H and Werre JM (1997*b*) Breast-cancer mortality in a non-randomized trial on mammographic screening in women over age 65. *Int J Cancer* **70**: 164–168

Elwood JM, Cox B and Richardson AK (1993) The effectiveness of breast cancer screening by mammography in younger women. *Online J Curr Clin Trials:* Doc No 32

Elston CE (1987) Grading of invasive carcinoma of the breast. In: *Diagnostic Histopathology of the Breast*, Page DL and Anderson TJ (eds), pp. 300–307. Churchill Livingstone: Edinburgh

Fracheboud J, Koning de HJ, Beemsterboer PM, Boer R, Hendriks JH, Verbeek AL, Ineveld van BM, Bruyn de AE and Maas van der PJ (1998) Nation-wide breast cancer screening in the Netherlands: results of initial and subsequent screening 1990–1995. National Evaluation Team for Breast Cancer Screening. *Int J Cancer* **75**: 694–698

Freund KM, Burns RB and Antab L (1998) Improving residents' performances of clinical breast examination. *J Cancer Educ* **13**: 20–25

Harris KM and Vogel VG (1997) Breast cancer screening. *Cancer Metastasis Rev* **16**: 231–262

Jannink I, Diest van PJ and Baak JPA (1995) Comparison of the prognostic value of four methods to assess mitotic activity in 186 invasive breast cancer patients. *Hum Pathol* **26**: 1086–1092

Linden van der HC, Baak JPA, Lindeman J, Hermans F and Meyer CLJM (1986) Morphometry and breast cancer. II. Characterisation of breast cancer cells with high malignant potential in patients with spread to lymph nodes: preliminary results. *J Clin Pathol* **39**: 603–609

Linden van der JC, Baak JPA, Lindeman J, Hermans J and Meyer CJLM (1987) Prospective evaluation of prognostic value of morphometry in patients with primary breast cancer. *J Clin Pathol* **40**: 302–306

Linden van der JC, Lindeman J, Baak JPA, Meijer CJLM and Herman CJ (1989) The multivariate prognostic index and nuclear DNA content are independent prognostic factors in primary breast cancer patients. *Cytometry* **10**: 56–61

Maestro C, Cazenave F, Marcy PY, Bruneton JN, Chauvel C and Bleuse A (1998) Systematic ultrasonography in asymptomatic dense breasts. *Eur J Radiol* **26**: 254–256

Peer PG, Verbeek AL, Straatman H, Hendriks JH and Holland R (1996) Age-specific sensitivities of mammographic screening for breast cancer. *Breast Cancer Res Treat* **38**: 153–160

Peeters PHM, Miltenburg GAJ, Fracheboud J, Gimbrère CHF, Hogervorst JMW and Colette HJA (1998) Seventeen-year evaluation of breast cancer screening: the DOM project, the Netherlands. *Br J Cancer* **78**: 962–965

Schroën AMA, Wobbes Th and Sluis van der RF (1996) Interval carcinomas of the breast: a group with intermediate outcome. *J Surg Oncol* **63**: 141–144

Schroën AMA, Wobbes Th and Sluis van der RF (1998) Infiltrating lobular carcinoma of the breast detected by screening. *Br J Surg* **85**: 390–392

Smart CR (1992) Mammographic screening: efficacy and guidelines. *Curr Opin Radiol* **4**: 108–117

Tabàr L et al (1985) Reduction in mortality from breast cancer after mass screening with mammography. Randomised trial from the Breast Cancer Screening Working Group of the Swedish National Board of Health and Welfare. *Lancet* **1**: 829–832

Theissig F, Baak JPA, Schuurmans L, Haroske G, Meyer W and Kunze KD (1996) Blind multicenter evaluation of the prognostic value of DNA image cytometric and morphometric features in invasive breast cancer. *Anal Cell Pathol* **10**: 85–99

Uyterlinde AM, Schipper NW and Baak JPA (1987) Comparison of extent of disease and morphometric and DNA flow cytometric prognostic factors in invasive ductal breast cancer. *J Clin Pathol* **40**: 1432–1436

Uyterlinde AM, Schipper NW, Baak JPA, Peterse H and Matze E (1988) Limited prognostic value of cellular DNA content to classical and morphometrical parameters in invasive ductal breast cancer. *Am J Clin Path* **89**: 301–307

Uyterlinde AM, Baak JPA, Schipper NW, Peterse H, Matze E and Meyer CJL (1990) Further evaluation of the prognostic value of morphometric and flow cytometric parameters in breast-cancer patients with long follow-up. *Int J Cancer* **45**: 1–7

Verbeek ALM, Hendriks JHCL, Holland R, Mravunac M, Sturmans F and Day NE (1984) Reduction of breast cancer mortality through mass screening with modern mammography. First results of the Nijmegen project, 1975–1981. *Lancet* **1**: 1222–1224

Wolfe JN (1991) Breast cancer screening. A brief historical review. *Breast Cancer Res Treat* **18**: 89–92

© 2000 Cancer Research Campaign
